# Global dissemination of conjugative virulence plasmids co-harboring hypervirulence and multidrug resistance genes in *Klebsiella pneumoniae*

**DOI:** 10.1128/msystems.01675-24

**Published:** 2025-03-25

**Authors:** Qi Xu, Ruanyang Sun, Xiaoxuan Liu, Heng Heng, Xuemei Yang, Miaomiao Xie, Chen Yang, Lianwei Ye, Edward Wai-Chi Chan, Rong Zhang, Sheng Chen

**Affiliations:** 1 State Key Laboratory of Chemical Biology and Drug Discovery and the Department of Food Science and Nutrition, The Hong Kong Polytechnic University, Kowloon, Hong Kong, China; 2 Department of Infectious Diseases and Public Health, Jockey Club College of Veterinary Medicine and Life Sciences, City University of Hong Kong, Kowloon, Hong Kong, China; 3 Department of Clinical Laboratory, School of Medicine, Second Affiliated Hospital of Zhejiang University, Hangzhou, Zhejiang, China; 4 Shenzhen Key Laboratory for Food Biological Safety Control, Food Safety and Technology Research Centre, The Hong Kong PolyU Shenzhen Research Institute, Shenzhen, Guangdong, China; Northwestern University Feinberg School of Medicine, Chicago, Illinois, USA

**Keywords:** *Klebsiella pneumoniae*, conjugative, hypervirulence, multidrug resistance, pVir-MDR

## Abstract

Carbapenem-resistant hypervirulent *Klebsiella pneumoniae* (CR-hv*Kp*) has led to a high mortality rate in the clinical setting and garnered significant attention in the clinical and scientific communities. It is still not clear which major mechanisms mediate the rapid evolution of hv*Kp* in clinical settings since the plasmid-encoding hypervirulence phenotype is considered non-conjugative. In this study, we revealed a conjugative plasmid, p16HN200-Vir, encoding virulence-associated *iuc* operon (*iucABCDiutA*) and resulting in a hypervirulent phenotype. *In silico* analysis of *Kp* strains from NCBI predicted a total of 94 p16HN200-Vir-like conjugative virulence plasmids. These sequences were identified in 19 sequence types (STs) of the host with significant geographical variations in distribution across countries and continents. Notably, ST11 was predominant in China, while ST147 and ST395 were prominent in the United Kingdom and Russia, respectively. These plasmids could be categorized into seven lineages, some of which formed distinct geographical clusters in China and the UK, while others exhibited a hybrid population. Importantly, most of the plasmids carried different carbapenemases, including *bl*a_NDM-1/5_ (lineage 6 and 7), and *bla*_OXA-48/232_ (lineage 2 and 5). Specifically, plasmids recovered from the United Kingdom and Russia were lineage-specific, with *bla*_NDM-1_ present in lineage 6 and *bla*_NDM-5_ in lineage 7. Our data suggest that this type of conjugative plasmids has spread around the world and contributed significantly to the current rapid evolution of CR-hv*Kp* in clinical settings. Further surveillance of these types of conjugative plasmids in clinical *Kp* is warranted.

**IMPORTANCE:**

In this study, we found that the conjugative virulence plasmids containing carbapenem resistance genes could be conjugated to *Klebsiella* strains, enabling them to express antibiotic resistance and hypervirulence-associated phenotypes simultaneously. More importantly, we observed that the global dissemination of this type of multidrug resistance and hypervirulence conjugative plasmid is lineage specific. The UMAP projection revealed that the regional variations were correlated with ST types and serotypes, which poses a global threat of the CR-hv*Kp* infection worldwide.

## INTRODUCTION

*Klebsiella pneumoniae* (*Kp*) is a common cause of hospital- and community-acquired infections, attracting public attention for its ability to acquire new plasmids and other mobile genetic elements carrying resistance- and/or virulence-associated genes (1–3). This pathogen can cause a wide spectrum of infections, including pneumonia, sepsis, urinary tract infections, and liver abscesses, among other pathologies (4). *Kp* was estimated to have contributed to 790,000 deaths worldwide, with 214,000 of those related to neonatal sepsis (5). *Kp* is listed in the latest WHO Bacterial Priority Pathogens List as a critical priority for which novel treatments are urgently needed (6). *Kp* strains are broadly categorized as classical *Kp* (c*Kp*) and hypervirulent *Kp* (hv*Kp*) (7), distinguished by disease profiles and genetic characteristics. c*Kp* is often found in healthcare settings and carries plasmid(s) coding for antimicrobial resistance, with sequence type (ST) 11 carbapenem-resistant *Kp* (CR-*Kp*) being the most prevalent strain in China. Hv*Kp* is currently the most common pathotype that can be differentiated from classic *Kp* (c*Kp*) due to the carriage of a cluster of virulence factors located in a virulence plasmid or other mobile genetic elements that can be integrated into the chromosome (8). Potential biomarkers, including *peg-344*, *iroB*, *iucA*, *rmpA,* and *rmpA2,* as well as increased siderophore production in hv*Kp*, were previously identified for the accurate differentiation of these two pathotypes (9).

The threat posed by hv*Kp* strains has been compounded by the fact that they undergo evolution continuously, rendering them resistant to carbapenems and various other antibiotics. Evidence gathered in our laboratory and others showed that the emergence of carbapenem-resistant hv*Kp* (CR-hv*Kp*) was due to the acquisition of a pLVPK-like virulence plasmid by the carbapenem-resistant strains (10–12) or plasmids containing various carbapenemase genes, such as *bla*_KPC-2_, *bla*_VIM_, or *bla*_NDM-1_, harbored by the hv*Kp* strains (13, 14). Studies focusing on the transmission of self-transferable carbapenem-resistant plasmids have provided powerful support for the possibility that hv*Kp* evolved into CR-hv*Kp* (2). However, the virulence plasmids of *Kp* are generally regarded as nonconjugative, and few studies have investigated the transfer of *Kp* virulence plasmids. Several recent studies have reported that a virulence plasmid could be conjugative when fused with a helper plasmid or KPC plasmid (11). We discovered a fusion virulence plasmid in a clinical *Klebsiella variicola* strain and confirmed its self-transferable ability (15). We previously observed homologous recombination between a virulence plasmid and IncFIA or IncFII type plasmids in a clinical *Kp* strain, resulting in a conjugative hybrid plasmid (11). Recently, the emergence of the large non-pLVPK-like virulence plasmid containing both MDR genes and conjugative transfer genes has been reported in many cases from 2016 to 2023 (16–26). Due to extremely difficult treatment, with an increase in the risk of mortality among patients, the convergence of antimicrobial resistance and virulence genes has become a major concern.

However, there have been no systematic studies exploring the molecular genetic signatures of Hv-MDR plasmid acquisition or elucidating the types, characteristics, and multiple antimicrobial resistance gene carriages of plasmids that co-harbor virulence genes, resistance genes, and conjugative transfer genes. In this study, we aimed to reveal the global emergence and dissemination of junction virulence plasmids carrying hypervirulence and multiple drug resistance genes by collecting complete plasmid sequences containing virulence determinants from the NCBI database and performing comparative analyses. Our results provide crucial insights for a more effective response to the risks associated with the evolution of Hv-MDR-bearing plasmids and for more precise prevention and control of the spread of these plasmids and bacterial resistance, which have significant public health implications.

## MATERIALS AND METHODS

### Bacterial strains and identification

*Kp* strain 16HN200 was isolated from a patient in a hospital located in Henan province, China, in 2016. The species identity of the strain was determined using the Vitek 2 system (bioMérieux, France) and confirmed by matrix-assisted laser desorption ionization-time of flight mass spectrometry apparatus (Bruker, Germany). Virulence factors (*rmpA*, *rmpA2*, *iucA*, and *iroN*) carried by this strain were detected through whole genome sequencing, and the string test was conducted on blood agar as previously described (10). All the bacterial culture, including conjugation assay, serum killing assay, and adherence and invasion assay were approved by the Hong Kong Polytechnic University and followed the guidelines on Biosafety in the Clinical Laboratory.

### Mice

Six- to eight-week-old animals of both sexes were used in this study. WT C57BL/6 mice were bred in-house or purchased from Cyagen. All mice were housed and bred under specific pathogen-free conditions. All experiments were performed using sex- and age-matched controls.

### Murine bacteremia models

*Kp* strain SH12019 and SH12019-TC were grown in LB broth for 18 h at 37°C. Cultures were then diluted at 1:100 and grown for an additional 2.5 h to reach the early logarithmic phase. Bacteria were pelleted by centrifugation and washed twice in cold phosphate-buffered saline (PBS) and then resuspended to achieve the desired density. Mice were infected with 1 × 10^4^ colony-forming units (CFU) of *Kp* strains intraperitoneally.

### Antibiotic susceptibility test

The antimicrobial susceptibility of strain 16HN200 and the related transconjugant strains was determined by the microdilution method according to the guidelines recommended by the Clinical and Laboratory Standards Institute (CLSI) (27) and Clinical Breakpoints and Guidance by the European Committee on Antimicrobial Susceptibility Testing (EUCAST) of 2021 (28). *Escherichia coli* strain 25922 served as a quality control strain. Antimicrobial agents tested included imipenem, ertapenem, meropenem, cefotaxime, ceftazidime, amikacin, ciprofloxacin, tigecycline, polymyxin B, and tellurite (Table 1). All tests were performed in duplicate, and each test included three biological replicates per strain.

**TABLE 1:** Phenotypic characteristics of carbapenem-resistant *Kp* strain 16HN-200 and the corresponding transconjugants^*a*^

Strain	IMP	ETP	MRP	CTX	CAZ	AMK	CIP	TIG	PB	TE
16HN200	>32	>32	>16	>128	64	>128	>32	4	2	128
EC600-TC	>32	>32	>16	>128	64	>128	>32	4	4	32
SH12019-TC	32	>32	>16	>128	>128	>128	>32	2	4	128
HvKP4-PC-TC	32	>32	>16	>128	128	>128	>32	2	4	>128
EC600	2	0.12	0.12	1	0.5	4	>32	4	2	2
HvKP4-PC	32	>32	>16	>128	64	>128	>32	2	32	2
SH12019	32	>32	>16	>128	64	>128	>32	2	2	2

^*a*^ IMP, imipenem; ETP, ertapenem; MRP, meropenem; CTX, cefotaxime; CAZ, ceftazidime; AMK, amikacin; CIP, ciprofloxacin; TIG, tigecycline; PB, polymyxin B; TE, tellurite.

### DNA sequencing and bioinformatics analysis

Genomic DNA was extracted using the PureLink Genomic DNA Mini Kit (Invitrogen, United States) according to the manufacturer’s instructions and subjected to whole genome sequencing using Illumina HiSeq (San Diego, CA, USA) and ONT MinION platform (Oxford, UK). Both short and long reads were *de novo* hybrid assembled using Unicycler v0.4.7 (29). Alignment of plasmids with similar structures was generated by BLAST Ring Image Generator (BRIG) v0.95 (30). Antimicrobial resistance genes and virulence factors were identified via a read-mapping approach with ABRicate v1.0.1 (https://github.com/tseemann/abricate), using publicly available ResFinder (31) and VFDB (32), respectively. Mobility prediction was recognized using MOB-suite v3.1.9. Assembled genome sequences were annotated with RAST v2.0 and Prokka v1.14.6 (33). Pan-genome was generated using Panaroo v1.2.10 (34). A phylogenetic tree was inferred from core genome alignment with RaxML v 8.2.12 (35) with 100 bootstrap replicates.

### Conjugation assay

For testing whether the virulence plasmid could undergo conjugation, rifampin-resistant *E. coli* strain EC600 was used as recipients. Strain 16HN200 and the recipient strains were cultured to optical density at 600 nm (OD_600_) = 0.6 at 37°C in LB medium. In the next step, 100 µL culture of the donor cells and 400 µL culture of the recipient cells were mixed and inoculated carefully into a 0.45 µm membrane placed on the surface of an LB agar plate. After incubation at 37°C overnight, bacteria on the membrane were collected, resuspended in saline, and serially diluted. The diluted culture was spread onto China Blue agar plates containing 2 µg mL^−1^ potassium tellurite (K_2_TeO_3_) and 600 µg mL^−1^ rifampin if strain EC600 was used as a recipient. The conjugation experiment was also performed by using *E. coli* transconjugant EC600-TC as the donor and classic CR-*Kp* strain as recipients. MacConkey agar plates containing 8 µg mL^−1^ potassium tellurite (K_2_TeO_3_) and 2 µg mL^−1^ meropenem were used to select transconjugants. The presence of *rmpA2* as a marker gene of virulence plasmid in transconjugants was determined by PCR as previously described (10).

### Mucoviscosity assay extraction and quantification of capsule

The mucoviscosity of the *Kp* strains SH12019 and SH12019-TC was determined by performing the sedimentation assay as previously described (36). Briefly, overnight culture grown in LB was diluted to an OD_600_ of 0.2 in media and grown at 37°C. At 6 h, the culture was normalized to an OD of 1.0 mL^−1^ and centrifuged for 5 min at 1,000 × *g*. The supernatant was removed without disturbing the pellet for OD_600_ measurement.

Uronic acid was extracted and quantified as described previously (37). Briefly, cultures were grown for 6 h as described above. Then, 500 µL culture was mixed with 100 µL capsule extraction buffer (100 mM citric acid, 1% Zwittergent 3-12), followed by incubation at 50°C for 20 min before centrifugation to pellet the cellular debris (5 min, 13,000 × *g*, room temperature). Capsule components were precipitated by incubating aliquots of supernatant (300 µL) with 1.2 mL absolute ethanol and centrifuged for 5 min at 13,000 × *g*. The pellet was air-dried and re‐suspended in 200 µL of sterile water, to which 1.2 mL of tetraborate solution (12.5 mM sodium tetraborate in sulfuric acid) was added and incubated for 5 min at 100°C, followed by immediate cooling on ice for at least 10 min. Uronic acid was detected by the addition of 20 µL of hydroxyphenyl reagent (0.15% 3‐phenylphenol in 0.5% NaOH). After a 5 min incubation at room temperature, the absorbance at 520 nm was measured. A standard curve constructed using glucuronic acid (Sigma-Aldrich) solutions of different concentrations was used to calculate the uronic acid concentration in the test samples. Results were presented as mean and standard deviation of data from three independent experiments.

### Serum killing assay

A serum bactericidal assay was performed to test the ability of bacterial strains to resist serum killing. Bacterial strains were cultured in LB broth at 37°C and harvested in the early logarithmic phase (OD_600_ = 0.3). Next, the bacterial suspension was adjusted to a concentration of 1  ×  10^8^ bacteria mL^−1^ in saline. A mixture of 50  µL of bacterial suspension and 150  µL of normal human serum was then incubated at 37°C in a 96-well plate (38). Viable bacterial counts were recorded at 0, 1, 2, and 3 h with duplications.

### Adherence and invasion assays

RAW 264.7 cells were grown in Dulbecco's modified Eagle medium (DMEM) supplemented with 10% heat-inactivated fetal bovine serum (FBS) and 1% nonessential amino acids (Gibco). The adherence assays and invasion assays of *Kp* were performed as described previously (37). In these assays, cells were seeded in 24-well plates overnight before *Kp* infection. Cells in 24-well plates (~5 × 10^5^ cells per well) were prewashed with PBS. Mid-log-phase *Kp* (OD_600_ = 0.4 to 0.6) in FBS-free DMEM was added to each well to achieve an MOI of 50. The number of CFUs inoculated per well was determined by serial dilution in PBS, plating on LB agar, and incubation for 12 h. For the adherence assay, the infected plates were centrifuged for 5 min at 200 × *g* prior to the incubation to promote adherence of bacteria to cells; the plates were then incubated for 30 min in a humidified 5% CO_2_ atmosphere at 37°C. The wells were then washed three times with PBS, and adherence of bacteria to the wells was disrupted by the addition of 1 mL 0.2% Triton X-100. The adherence rate was the proportion of the inoculum that adhered to the wells of the plate.

To perform invasion assays, *Kp* in DMEM medium containing 10% FBS was added to the wells, incubated for 2 h, and washed three times with PBS, followed by a second incubation for 2 h with fresh medium containing 300 µg mL^−1^ amikacin. Finally, the number of putative viable intracellular bacteria was determined by plating serial dilutions of the disrupted mixture onto LB agar and incubated for 12 h at 37°C. The invasion rate was the proportion of the inoculum that was internalized.

### Conjugative virulence plasmid alignment from the database

We used the plasmid (MW911669.1) in the PLSDB set as the references for BLASTN and used a length of 2,000 bp, coverage of 80%, identity of 80%, and overall plasmid coverage of 60% as the thresholds to define a plasmid hit for genomes collected from the NCBI Pathogen database (https://www.ncbi.nlm.nih.gov/pathogens/) access in October 2023. MOB-suite v 3.1.9 was applied to reconstruct plasmids from draft assemblies. Similar plasmids were extracted from the assemblies and annotated using Prokka v1.14.6. Pan-genome was generated using Panaroo v1.2.10 and visualized with the UMAP algorithm.

### Statistical analysis

Statistical analysis of data obtained in this work was performed by means of using GraphPad Prism 6.0 (GraphPad Software, La Jolla, CA, USA, www.graphpad.com). Statistical analyses on normally distributed data sets were performed using one-way ANOVA with Tukey’s correction for multiple comparisons. The log-rank test was used for comparing survival rates in animal experiments. *P* values < 0.05 were considered significant. Unless otherwise indicated, the survival curve of mice in animal experiments and results of flow cytometry analysis were representative of at least two independent experiments.

## RESULTS

### The characterization of plasmid co-harboring MDR and virulence genes

**Fig 1:**
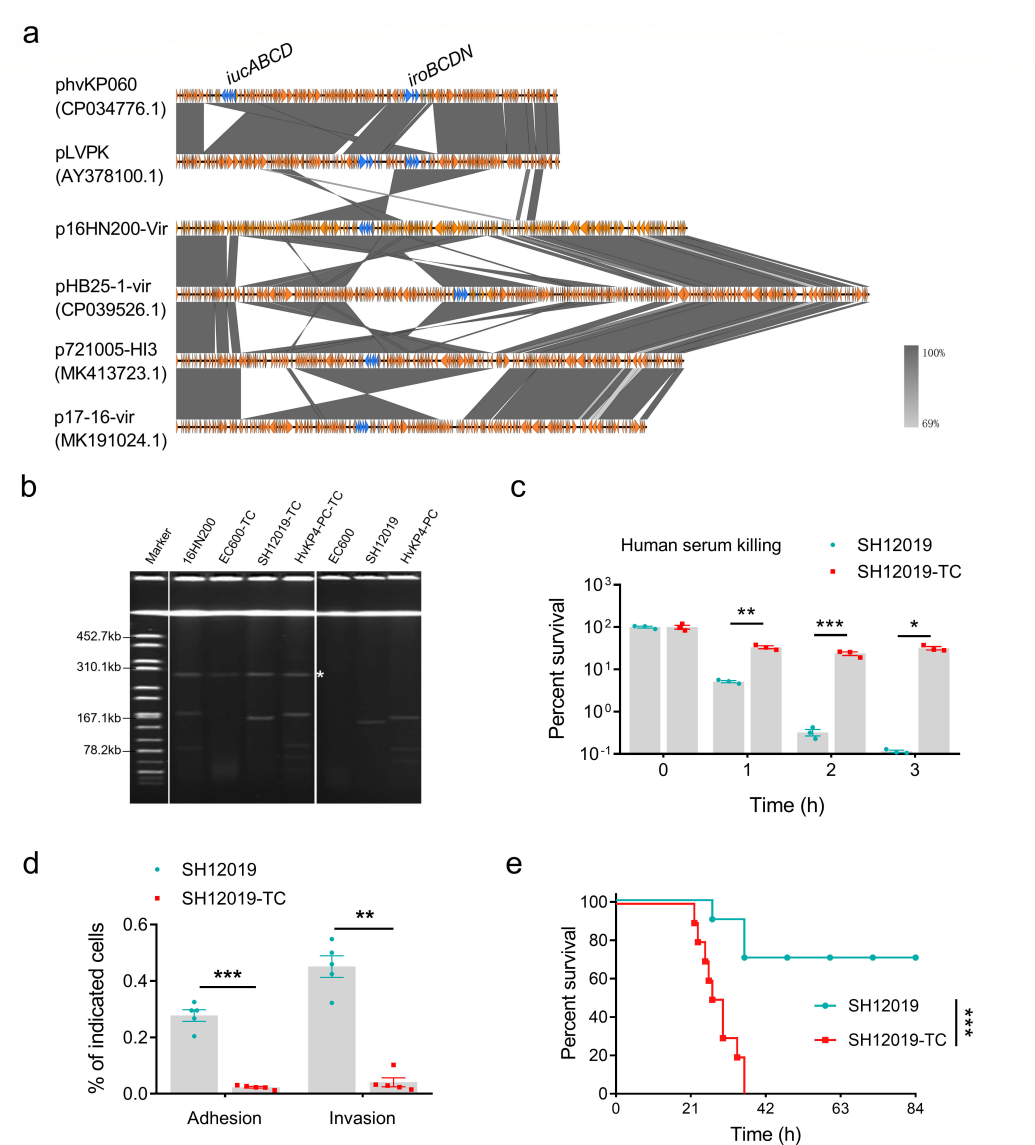
Transferability and virulence of p16HN200-Vir. (a) Alignment of p16HN200-Vir with plasmids of similar structures using EasyFig. (b) Transconjugant EC600-TC is a transconjugant obtained by conjugation using strain 16HN200 as the donor and *E. coli* strain EC600 as the recipient strain. Transconjugants SH12019-TC and HvKP4-PC-TC were generated using EC600-TC as the donor and SH12019 and HvKP4-PC as recipient strains, respectively. * indicates the conjugative virulence plasmid. (c) The resistance of *Kp* strains to killing by pooled human serum. (d) Assay of the ability of *Kp* strains to adhere to and invade RAW264.7 cells. Data were analyzed using one-way ANOVA test. Each data point was repeated three times. Data are presented as the mean ± SEM. (e) Virulence potential of *Kp* strains in the mouse bacteremia infection model infected with 5 × 10^7^ of the test strains. The survival of mice infected by each test *Kp* strain is depicted. The test strains included SH12019 and SH12019-TC. *n* = 10. A log-rank (Mantel–Cox) test conducted for the survival curves revealed significant differences. *P < 0.05; **P < 0.01; ***P < 0.001; ****P < 0.0001.

In this study, we identified a 292,353 bp, non-pLVPK-like IncFIB/IncHI1B plasmid (p16HN200-Vir) from a clinical carbapenem-resistant *Kp* strain. This plasmid co-harbors multiple virulence factors, including the regulator of mucoid phenotype 2 (*rmpA2*), aerobactin (*iucABCD*, *iutA*), and antibiotic resistance genes, e.g., *qnrB4*, *bla*_DHA-1_, *sul1*, *msr*(E), *mph*(E), *armA*, *bla*_SHV-11_, and *mph*(A). Using BLAST against the National Center for Biotechnology Information (NCBI) database, this plasmid was found to show highest similarity (90% coverage and 99% identity) to plasmid p17-16-vir (MK191024.1), a 290,451 bp IncFIB/IncHI1B plasmid recovered from a *Kp* strain isolated from sputum in 2017 in China (17). Plasmid p16HN200-Vir shared a 65-kbp insertion region from virulence plasmid pLVPK, also including the tellurium resistance cluster, *rmpA2* and *iucABCDiutA* genes (Fig. 1a). These genetic changes indicated the adaptation of virulence plasmids with *Kp* strains during the transmission process. These virulence plasmids lacked virulence genes *rmpA* and *iroBCDN* when compared with typical virulence plasmid pLVPK, which may affect the virulence potentials of these CR-*Kp* strains. Meanwhile, plasmid p16HN200-Vir harbors the plasmid transfer *tra* gene cluster.

We performed a conjugation experiment to confirm if the *tra* gene carried by p16HN200-Vir could mediate conjugation for the virulence plasmids. We found that the virulence plasmid p16HN200-Vir could be conjugated to *E. coli* EC600 (Fig. 1b). Then, conjugation experiments were performed using EC600-TC as the donor strain and ST11 *Kp* strains SH12019 and HvKP4-PC as the recipient. Our data showed that the fusion plasmid could be conjugated to strain SH12019 and HvKP4-PC, resulting in SH12019-TC and HvKP4-PC-TC with a conjugation efficiency of 10^−4^ and 10^−5^, respectively (Fig. 1b). These data suggested that the conjugative virulence plasmid could be conjugated to clinical *Kp* strains.

To determine if the acquisition of p16HN200-Vir could confer phenotypic hypervirulence in *Kp*, transconjugants and control strains were subjected to assessment of their virulence potential using various biological assays. Serum resistance results showed that the survival rate of SH12019-TC was higher than that of SH12019, indicating that p16HN200-Vir confers high-level serum resistance in *Kp* (Fig. 1c), although transconjugants SH12019-TC, which had acquired the virulence plasmid, exhibited no significant difference in mucoviscosity compared with SH12019 (Fig. S1). Furthermore, SH12019-TC were more resistant to adhere and uptake by RAW 264.7 when compared with SH12019 (Fig. 1d). Furthermore, infection of mice with 5 × 10^7^ CFU of SH12019-TC led to 100% mortality at 36 h, compared with ~70% survival at 84 h for SH12019 (Fig. 1e), suggesting that acquisition of conjugative virulence plasmid resulted in an increase in the virulence level of SH12019. Strain HvKP4-PC-TC also showed comparable virulence to HvKP4-PC (Fig. S2). Together, we conclude that the virulence plasmid can confer virulence phenotype in recipient strains.

### Diversity and complexity of pVir-MDR host distribution

**Fig 2:**
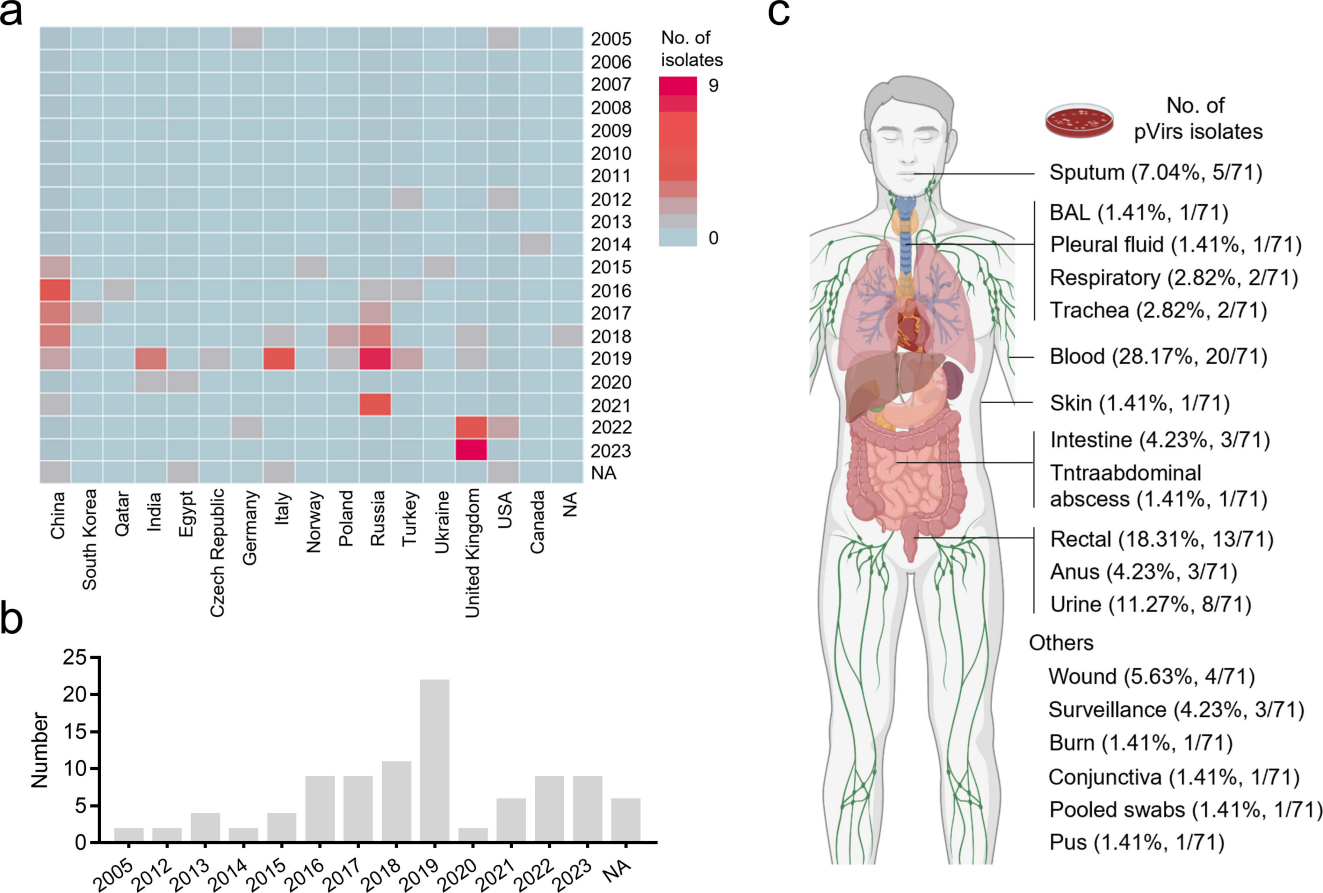
Temporal, geographic distribution, and isolation source of pVir-MDRs. (a) Heatmap of the pVir-MDRs detected in different countries in corresponding years. (b) Temporal distribution of pVir-MDRs. “NA” indicated that the relative information was not provided. (c) Isolation source of the pVir-MDRs.

As the emergence of the large non-pLVPK-like virulence plasmid containing both MDR genes and conjugative transfer genes has been reported recently, we were curious about whether these plasmids shared features for the prevalence of this kind of plasmid. We conducted a comprehensive analysis of complete conjugative plasmids in the NCBI GenBank database and isolates from our own collection to confirm the prediction. A total of 94 complete p16HN200-Vir-like plasmid sequences were collected from 16 countries between 2005 and 2023 (Fig. 2a). Since most of these plasmids carried virulence-encoding genes as well as antimicrobial resistance genes, we therefore renamed these plasmids as pVir-MDRs (Table S1). Among these plasmids, two of them were identified as early as 2005 in Germany and the USA, respectively. The virulence plasmid 371661_p1 (CP132674.1, 330,595 bp) harboring strain 371661 belonged to ST3586 and was isolated from a respiratory sample in Germany. The plasmid 5881 p1 (CP132710.1, 322,633 bp) harboring strain 5881 belongs to ST48 and was isolated from a wound in Washington, DC, USA. After that, an increasing number of strains harboring pVir-MDRs were isolated. Surprisingly, 22 strains containing this type of plasmid were collected in eight countries in 2019 (Fig. 2b). For the geographic origins of plasmids, China (26, 27.7%), Russia (19, 20.2%), and the United Kingdom (15, 16.0%) accounted for the top three countries.

In total, these plasmids were harbored by 19 ST types of *Kp*, the most common being ST147 (21, 22.3%), ST11 (12, 12.8%), ST395 (12, 12.8%), and ST15 (10, 10.6%) (Fig. S3a). The geographical distribution of STs was diverse, with significant differences across countries and continents in terms of the prevalent types. ST11 was the most prevalent type in Asia, while ST147 was the dominant type in Europe. In terms of country, ST11 was the dominant genotype in China; ST147 dominated in Italy, the United Kingdom, and the United States. In Russia, ST395 was the predominant ST type. Meanwhile, ST2906 was widespread in Turkey and India, and ST23 was mainly distributed in Poland (Fig. S3b). Among these pVir-MDRs, the isolation source of 71 plasmids was provided. The majority of the isolates were from blood (28.17%, 20/71), rectal (18.31%, 13/71), and urine (11.27%, 8/71), followed by sputum (7.04%, 5/71) (Fig. 2c). Taken together, these data indicate the diversity of temporal, geographic distribution, and isolation source of the pVir-MDRs.

### The genetic structure and diversity of ARGs carried by the pVir-MDRs

**Fig 3:**
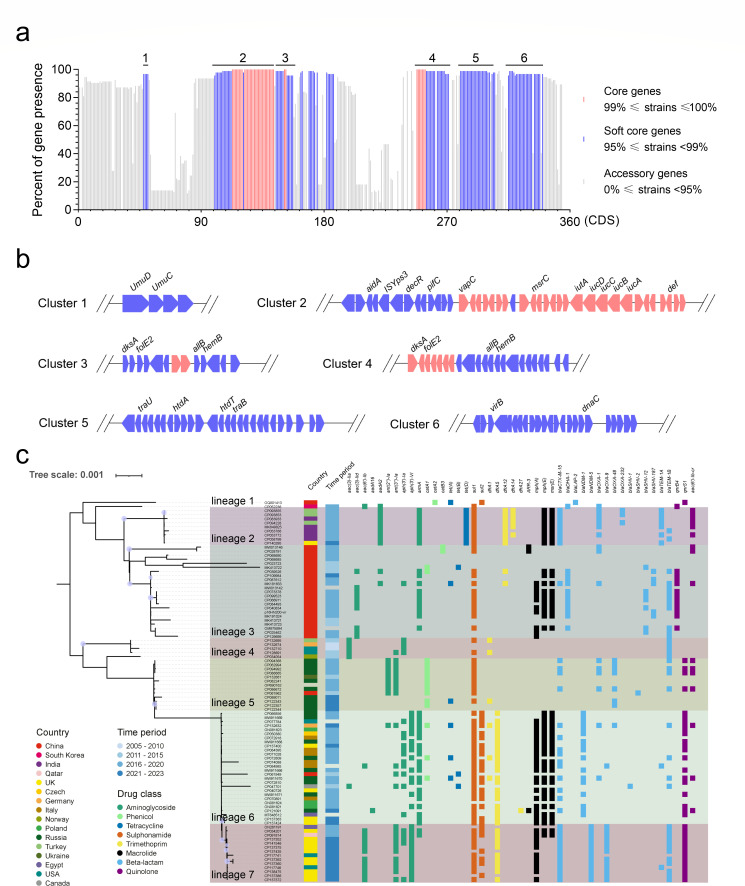
Phylogenetic analysis. (a) Graph of the percentage of gene presence within 94 pVir-MDRs. The presence of these genes more than 99% was considered as core genes, and presence between 95% and 99% was considered as soft core genes. The core genes and soft core genes were marked in light red and light blue, respectively. CDS: coding sequence. (b) The gene structure of six core gene clusters in pVir-MDRs as indicated in (a). The core genes and soft core genes were marked in light red and light blue, respectively. (c) Maximum-likelihood core genome phylogeny of 94 pVir-MDR plasmids. The isolation sources, collection years, and antimicrobial resistance genes are shown. Different classes of resistance genes were divided into different colored squares.

To better understand the structure of pVir-MDRs, pan-genome analysis was performed. We considered the presence at >99% as core genes and presence between 95% and 99% as soft core genes. Among these 329 genes, 39 genes were identified as core genes, and 114 genes were identified as soft core genes (Fig. 3a). The vast majority of genes annotated in each plasmid were hypothetical proteins. The virulence gene cluster *iucABCDiutA* was highly conserved and identified as core genes. The plasmid transfer genes *traU* and *traB* were identified as soft core genes (Fig. 3b). Together, these data suggest that virulence genes and *tra* transfer genes are highly conserved in pVir-MDRs, which confers this type of plasmid with both high virulence and transfer ability.

To elucidate the genetic relationship of pVir-MDRs, phylogenetic analyses were constructed using the core genes of 94 plasmids. The 94 plasmids could be classified in six lineages according to the topology of the phylogenetic tree (Fig. 3c). The clustering of pVir-MDRs was correlated with their geographic locations. The plasmid p16HN200-Vir was found to be located in a specific lineage (lineage 2) with these pVir-MDRs isolated from China throughout 2013–2021, demonstrating that the Chinese plasmids formed a local lineage distinct from representative plasmids of diverse geographical regions and that long-term local reservoirs have been established.

Next, we analyzed the antimicrobial resistance gene (ARG) carried by the pVir-MDRs, revealing that eight types of ARGs were harbored by these plasmids (Table S2). Antimicrobial resistance in *Kp* can be explained by the enzymatic breakdown of antibacterial drugs, as in the case of lactam resistance due to lactamases. Particularly, β-lactams are the most extensively used antibiotics, which include natural and synthetic penicillins and their derivatives, such as cephalosporins, cephamycins, monobactams, and carbapenems (39). These plasmids harbored several types of β-lactamases, including *bla*_CTX-M_, *bla*_DHA_, *bla*_LAP_, *bla*_NDM_, *bla*_OXA_, *bla*_SHV_, and *bla*_TEM_ (Table 2). The *bla*_DHA-1_ was mainly harbored by lineage three plasmids isolated from China. The *bla*_LAP-2_ only exists in pWYKP586-1 (OQ801413), a plasmid harboring by an ST11 *Kp* strain isolated in Shanghai. Notably, the *bla*_OXA-48_ was mainly harbored by lineage 5 plasmids from *Kp* strains mainly isolated from Russia (Fig. S4a). The *bla*_NDM-1_ and *bla*_NDM-5_ were only detected in special lineage 6 and lineage 7, respectively. The gene background (10 kbp upstream and downstream) of *bla*_NDM-1_ and *bla*_NDM-5_ was found to be conserved (Fig. S4b and c). The main elements involved in the capture and transfer of *bla*_NDM-5_ genes upstream and downstream were IS*Spu2*, IS*26*, IS*Aba125,* and IS*Shes11*, suggesting the popular and regional dissemination of the *bla*_NDM-5_ in these countries (Fig. S4b). The *bla*_NDM-1_ bearing plasmid p1, harbored by a ST395 *Kp* strain 110821 recovered from surveillance in Germany, contained *peg-344*, *rmpADC*, *rmpA2,* and *iucABCDiutA* (Fig. S4c). These biomarkers are linked on the canonical hv*Kp* virulence plasmid pLVPK (40), which has been shown to be critical for the expression of the hv*Kp* hypervirulent phenotype (41). For these two plasmids recovered in 2005, plasmid 371661 p1 (CP132674.1) and plasmid 5881 p1 (CP132710.1), both collected from *Homo sapiens*, shared similar structures and only harbored *aac(3)-Ila*, *aph(3’)-VI*, and *bla*_TEM-1A_ (Fig. 3c; Fig. S4d). It should be noted that these two conjugative pVir-MDRs were recovered only 2 years after the traditional pLVPK was reported, suggesting that the evolution of the hv*Kp* virulence plasmid was rapid.

**TABLE 2:** Detailed information of the β-lactamase types on pVir-MDRs

Number of plasmids	β-Lactamase types	Country
40.4%, 38/94	*bla*_CTX-M-15_	China (1), Egypt (1), Germany (1), India (5), Italy (6), Qatar (1), Russia (6), Turkey (3), UK (12), the USA (2)
13.8%, 13/94	*bla*_DHA-1_	China (12), South Korea (1)
1.1%, 1/94	*bla*_LAP-2_	China (1)
18.1%, 17/94	*bla*_NDM-1_	Czech (1), Egypt (1), Germany (1), Italy (1), Poland (2), Russia (9), UK (1), the USA (1)
14.9%, 14/94	*bla*_NDM-5_	Egypt (1), Qatar (1), UK (10), the USA (2)
17.0%, 16/94	*bla*_OXA-1_	China (3), Germany (1), India (3), Russia (5), South Korea (1), Turkey (3)
3.2%, 3/94	*bla*_OXA-232_	Turkey (3)
8.5%, 8/94	*bla*_OXA-48_	Germany (1), Russia (7)
14.9%, 14/94	*bla*_OXA-9_	Egypt (1), Italy (1), Qatar (1), UK (10), the USA (1)
7.4%, 7/94	*bla*_SHV-12_	China (7)
5.3%, 5/94	*bla*_SHV-187_	China (5)
1.1%, 1/94	*bla*_SHV-1_	Canada (1)
1.1%, 1/94	*bla*_SHV-2_	China (1)
9.57%, 9/94	*bla*_TEM-1A_	India (3), Italy (1), Turkey (3), UK (1), the USA (1)
40.4%, 38/94	*bla*_TEM-1B_	Canada (1), China (11), Egypt (1), Germany (2), India (2), Norway (1), Qatar (1), Russia (6), Turkey (1), UK (10), the USA (2)

### Genotypic assessment of the hv*Kp* cohorts

**Fig 4:**
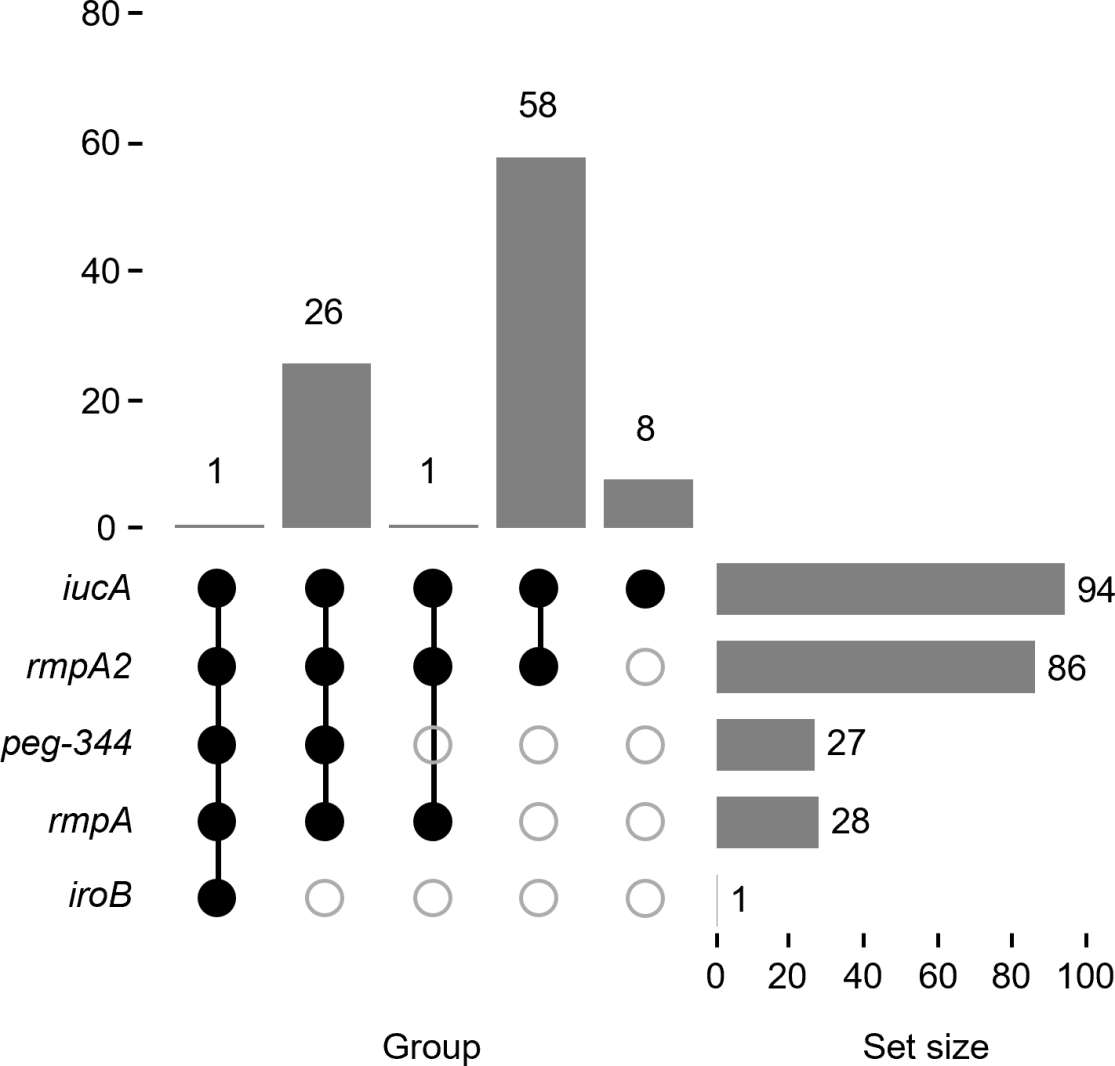
Virulence plasmid biomarker distribution. An UpSet graph for the 94 pVir-MDRs was generated to facilitate the visualization of the presence or absence of the biomarkers *iucA*, *iroB*, *peg-344*, *rmpA*, and *rmpA2*. Black circles designate the presence of a given marker, whereas gray circles designate its absence. The number above each bar represents the number of strains that possess that marker configuration.

Previous studies demonstrated that the presence of *iucA*, *iroB*, *peg-344*, *rmpA*, and *rmpA2*, which are linked on the hv*Kp*-specific virulence plasmid, possessed a > 95% accuracy for differentiating the hv*Kp*-rich and c*Kp*-rich strain cohorts (9). We next assessed all 94 pVir-MDRs for these genes. The hv*Kp* strain cohort has a significantly higher marker count (some combination of *iucA*, *iroB*, *peg-344*, *rmpA*, and *rmpA2*) compared with the c*Kp* strain cohort (9). Of note, only one unnamed plasmid (CP023723.1) isolated from Taiwan, China, possessed all five biomarkers. However, previous studies reported that only one or two markers of these five markers could be categorized as c*Kp* based on the murine infection model (9, 42). Of these, 58/94 (61.70%) possessed *iucA* and *rmpA2,* and 8/94 (8.51%) possessed *iucA* alone (Fig. 4; Table S1).

To further confirm this categorization, we compare the provided virulence potential of these pVir-MDRs. Strain SZS128 (*iucA* and *rmpA2* harbored by pSZS128-Hv-MDR, CP099523.1) exhibited lower virulence than the hv*Kp* control strain NTUH-K2044 but higher than QD110 (18). *E. coli* J53 harboring p1864-1 (*iucA* and *rmpA2*, CP084493.1) showed increased virulence compared with the recipient strain J53 (21). The virulence plasmid pSH12_Vir (*iucA* and *rmpA2*, CP040834.1) was also reported to play an important role in determining virulence of strain SH12 (17). However, *Galleria mellonella* larval and murine infection models indicated that no significant difference was found for XHKPN083 (*iucA* and *rmpA2*, CP066911.1) compared with XHKP75 (c*Kp*) (43). Together, these results suggested that the presence of virulence genes did not fully predict the pathogenicity of *Kp* strains.

### pVir-MDR population structure analysis

**Fig 5:**
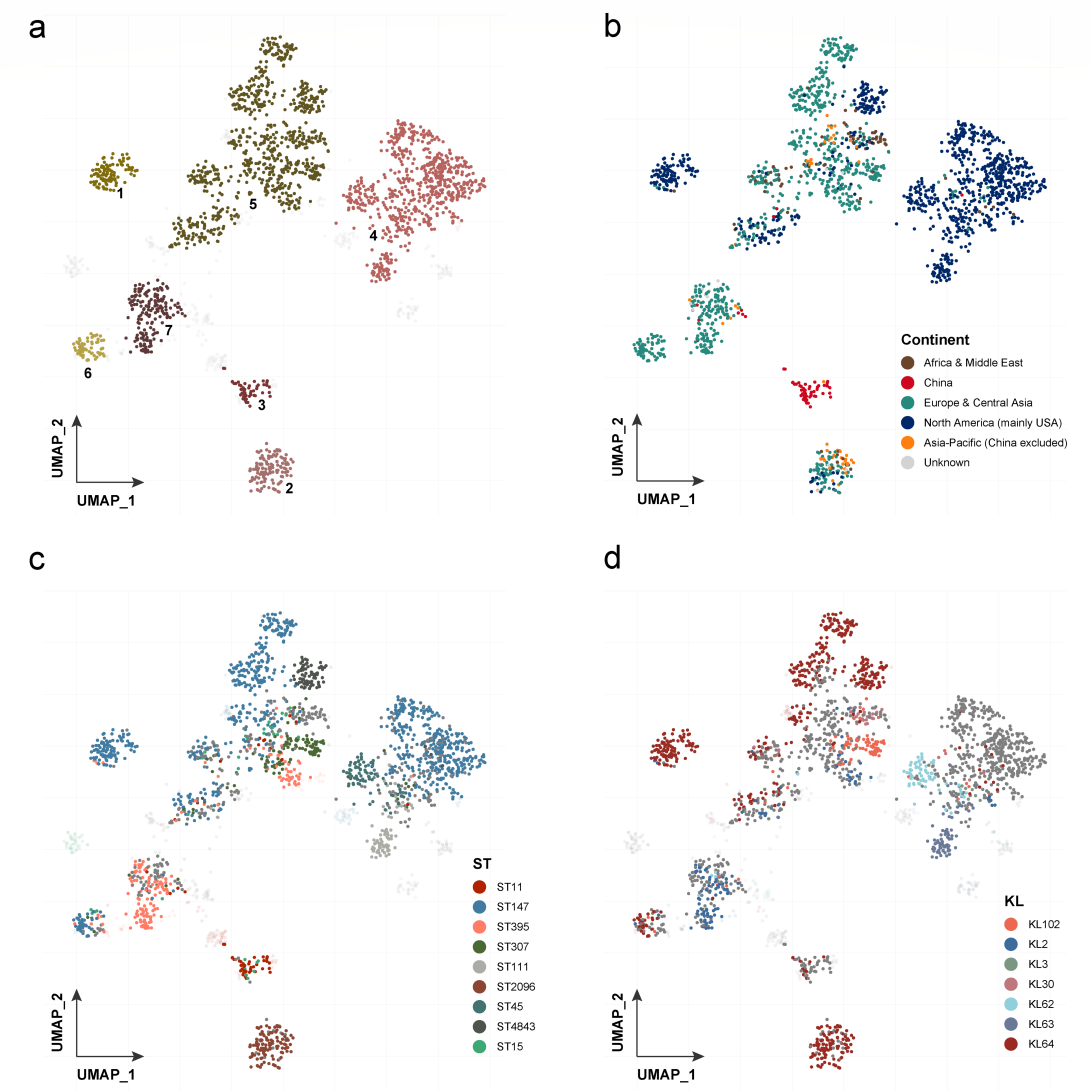
A UMAP projection of distances (measured by gene presence/absence) between the pVir-MDR plasmid. These are colored to show the variability explained by clustering (a), continent (b), ST type (c), and KL type (d).

The UMAP projection offers a comprehensive visualization of genetic distances between strains containing pVir-MDR plasmids according to the alignment results (44). This analysis reveals distinct patterns and clusters, providing insights into the genetic diversity and distribution of these plasmids across various dimensions. The presence of well-defined clusters suggests prevalent genetic configurations, potentially due to evolutionary pressures or horizontal gene transfer, highlighting potential evolutionary trajectories (Fig. 5a). The UMAP visualization also reveals significant geographic variability in plasmid composition. Plasmids from North America and Europe predominantly cluster together, indicating shared genetic traits possibly influenced by similar healthcare practices or ecological factors. In contrast, plasmids from Asia-Pacific and Africa show distinct clustering, reflecting regional variations (Fig. 5b). Additionally, the analysis identifies associations between specific sequence types (STs) and genetic configurations of the pVir-MDR plasmid. Notably, ST11 and ST2906 are prominently clustered, and ST147 is spread worldwide , indicating a strong correlation with particular genetic traits (Fig. 5c). Furthermore, serotypes KL102 and KL64 exhibit distinct clustering, suggesting a potential link between serotype and plasmid genetic composition (Fig. 5d). By integrating information on clustering, geographic distribution, sequence type, and serotype, the figure provides a comprehensive overview of the factors influencing plasmid diversity and dissemination. This multidimensional analysis enhances our understanding of the evolutionary dynamics and epidemiological patterns of plasmid-mediated multidrug resistance, informing strategies for surveillance, prevention, and control of resistant infections.

## DISCUSSION

We observed that all the pVir-MDRs contain *iucABCD* and *iutA*; however, only 27 (28.7%) of them harbor *peg-344* and *rmpA*, and only one (1.1%) harbors all five virulence biomarkers. As shown in Table S1, we noticed that most of the virulence biomarker *rmpA2* had base substitution, insertion, or deletion at different sites resulting in the frameshift or missense variants. The hypermucoviscous (HMV) phenotype of hv*Kp* usually results from *rmpA* and *rmpA2* located on the hypervirulence plasmid (7). Although a previous study found a significant correlation between the *rmpA* gene and HMV, it could not clarify why some *rmpA*-positive isolates did not demonstrate the HMV phenotype (45). However, it was later revealed that the absence of HMV and reduced virulence in some *rmpA*-positive *Kp* strains were caused by sequential mutations in the *rmpA* and *rmpA2* genes (46). Meanwhile, other studies reported some MDR-hv*Kp* isolates that lacked both *rmpA* and *rmpA2* biomarkers but carried other virulence factors, such as aerobactin and salmochelin, and still exhibited the HMV phenotype (47, 48). These studies indicated that MDR-hv*Kp* strains lacking both hyper-mucoid regulators (*rmpA/rmpA2*) but showing the HMV phenotype possessed the *iroE* and *iroN* (salmochelin) and *iutA* (aerobactin) genes (48, 49).

Hv*Kp* strains produce siderophore proteins, such as enterobactin, salmochelin, yersiniabactin, and aerobactin. The production of aerobactin by the *iucABCDiutA* cluster is particularly important in the context of host–pathogen interactions. By sequestering iron, aerobactin not only supports bacterial proliferation but also contributes to immune evasion. The ability to thrive in iron-restricted environments allows bacteria to colonize diverse tissues, leading to more severe and widespread infections. Furthermore, the presence of the *iucABCDiutA* cluster has been associated with increased virulence in clinical isolates of *Kp* (50) and *E. coli* (38). Experimental studies have indicated that aerobactin is the predominant contributor to hypervirulence and is more specific to hv*Kp* compared with enterobactin and yersiniabactin (51). Therefore, the presence of aerobactin biosynthesis genes (*iucABCD* and *iutA*) is considered a reliable biomarker for hv*Kp* (9). It is undoubtedly proven that the regulator of the mucoid phenotype genes (*rmpA* and *rmpA2*), along with siderophore-coding genes (aerobactin and salmochelin), altogether play a critical role in the HMV of *Kp* strains. However, the relationship among these genes, the HMV phenotype, and relevant virulence remains a mystery that requires further investigation.

In a previous study, the first conjugative virulence plasmid, p15WZ-82 Vir, was identified in *Klebsiella variicola*, demonstrating its capacity for self-transduction (15). Similarly, Tian et al. described the virulence plasmid pK2606 in *Kp*, which comprised a complete gene cluster for the type IV secretion system along with the *iucABCD-iutA* genes encoding aerobactin (52). To date, the emergence of conjugative virulence plasmids that also encode antimicrobial resistance or multidrug resistance (MDR) remains uncommon. Zhou et al. described the conjugative plasmid p1864-1 of the IncFIB/IncH1B type, which harbored both resistance genes and the *iuc* operon (21). Many studies have suggested a close relationship between the IncF subtype IncFIB/IncFII plasmids and antimicrobial resistance (AMR) genes (53, 54), whereas a recent study showed that the IncFIB(K) replicon significantly promotes the development and replication of virulence plasmids, especially those carrying the plasmid-borne *iuc* genes typically harbored by an IncFIB(K) replicon (55). Similarly, the formation mechanism of the pCY814036-iucA-like plasmid involved the acquisition of *iucABCD-iutA* by the IncFIB(K)/IncFII(K) conjugative plasmid (55). A striking finding is that similar virulence plasmids have been transferred between *Kp* strains of different sequence types (STs) (55). Furthermore, a linear alignment showed that variations between p16HN200-Vir and other similar plasmids were situated in the MDR region, indicating that drug resistance in these plasmids is continuously evolving via transposition or recombination. Although data from previous studies are still insufficient to fully characterize the MDR region harbored by p16HN200-Vir, studies have indicated that the active transmission of resistance genes has prompted the evolution of non-conjugative plasmids into conjugative MDR-hypervirulence plasmids (15, 56), implying the acquisition of drug resistance genes via homologous recombination. Recombination events mediated by insertion sequences might be responsible for the convergence of plasmids carrying MDR and virulence factors, raising significant concerns about the occurrence and spread of these plasmids and the effectiveness of current protocols for controlling such *Kp* strains.

## Data Availability

The complete sequence of plasmid p16HN200-Vir has been deposited in the GenBank database under accession number CP157746.1. All data and materials presented in this study are available on request.

## SUPPLEMENTAL MATERIAL

Supplemental material msystems.01675-24-s0001.docx Supplemental table captions and Fig. S1 to S4.

Table S1 msystems.01675-24-s0002.xlsx Detailed information, virulence biomarkers, and plasmid mobility of pVir-MDRs.

Table S2 msystems.01675-24-s0003.xlsx The presence of antimicrobial resistance genes in pVir-MDRs.

ASM does not own the copyrights to Supplemental Material that may be linked to, or accessed through, an article. The authors have granted ASM a non-exclusive, world-wide license to publish the Supplemental Material files. Please contact the corresponding author directly for reuse.
