# Supplementary material for: Global dissemination of conjugative virulence plasmids co-harboring hypervirulence and multidrug resistance genes in Klebsiella pneumoniae
Source: mSystems. 2025 Mar 25;10(4):e01675-24. doi: 10.1128/msystems.01675-24 (PMC12013265; doi:10.1128/msystems.01675-24)
Supplement: Supplemental material — Supplemental table captions and Fig. S1 to S4. [file msystems.01675-24-s0001.docx]

Supplementary Materials

**Global dismission of conjugative virulence plasmid coharboring hypervirulence and multidrug resistance genes in *Klebsiella pneumoniae***

Qi Xu^a,1^, Ruanyang Sun^a,1^, Xiaoxuan Liu^a,c^, Heng Heng^a,c^, Xuemei Yang^a^, Miaomiao Xie^a^, Chen Yang^a,c^, Lianwei Ye^a,c^, Edward Wai-Chi Chan^a^, Rong Zhang^d^, Sheng Chen^a,b*^

^a^ State Key Laboratory of Chemical Biology and Drug Discovery and the Department of Food Science and Nutrition, The Hong Kong Polytechnic University, Kowloon, Hong Kong, China

^b^ Department of Infectious Diseases and Public Health, Jockey Club College of Veterinary Medicine and Life Sciences, City University of Hong Kong, Kowloon, Hong Kong, China

^c^ Department of Clinical Laboratory, Second Affiliated Hospital of Zhejiang University, School of Medicine, Zhejiang, Hangzhou, China

^d^ Shenzhen Key Laboratory for Food Biological Safety Control, Food Safety and Technology Research Centre, The Hong Kong PolyU Shenzhen Research Institute, Shenzhen, People’s Republic of China

^1^ These authors contribute equal.

*To whom correspondence should be addressed: Dr. Sheng Chen, Department of Food Science and Nutrition, The Hong Kong Polytechnic University, Hom Hung, Kowloon, Hong Kong, China. Email: sheng.chen@polyu.edu.hk; Tel: +852 3400-8619.

**Legends for Supplementary Table**

**Table S1. The list of detailed information, virulence biomarkers and plasmid mobility of pVir-MDRs.**

**Table S2.** **The presence of antimicrobial resistance genes in pVir-MDRs.**

**
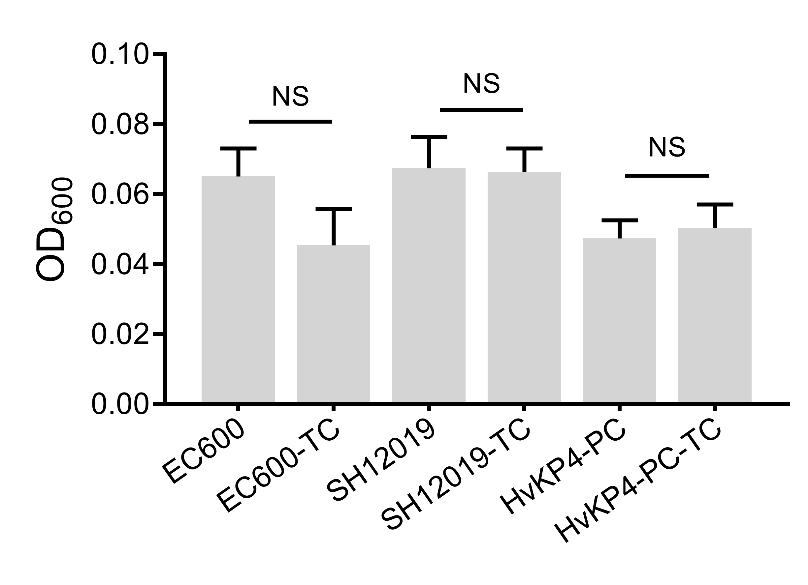
**

**Figure S1. The mucovisicosity assay between recipient strains and its transconjugants.**

**
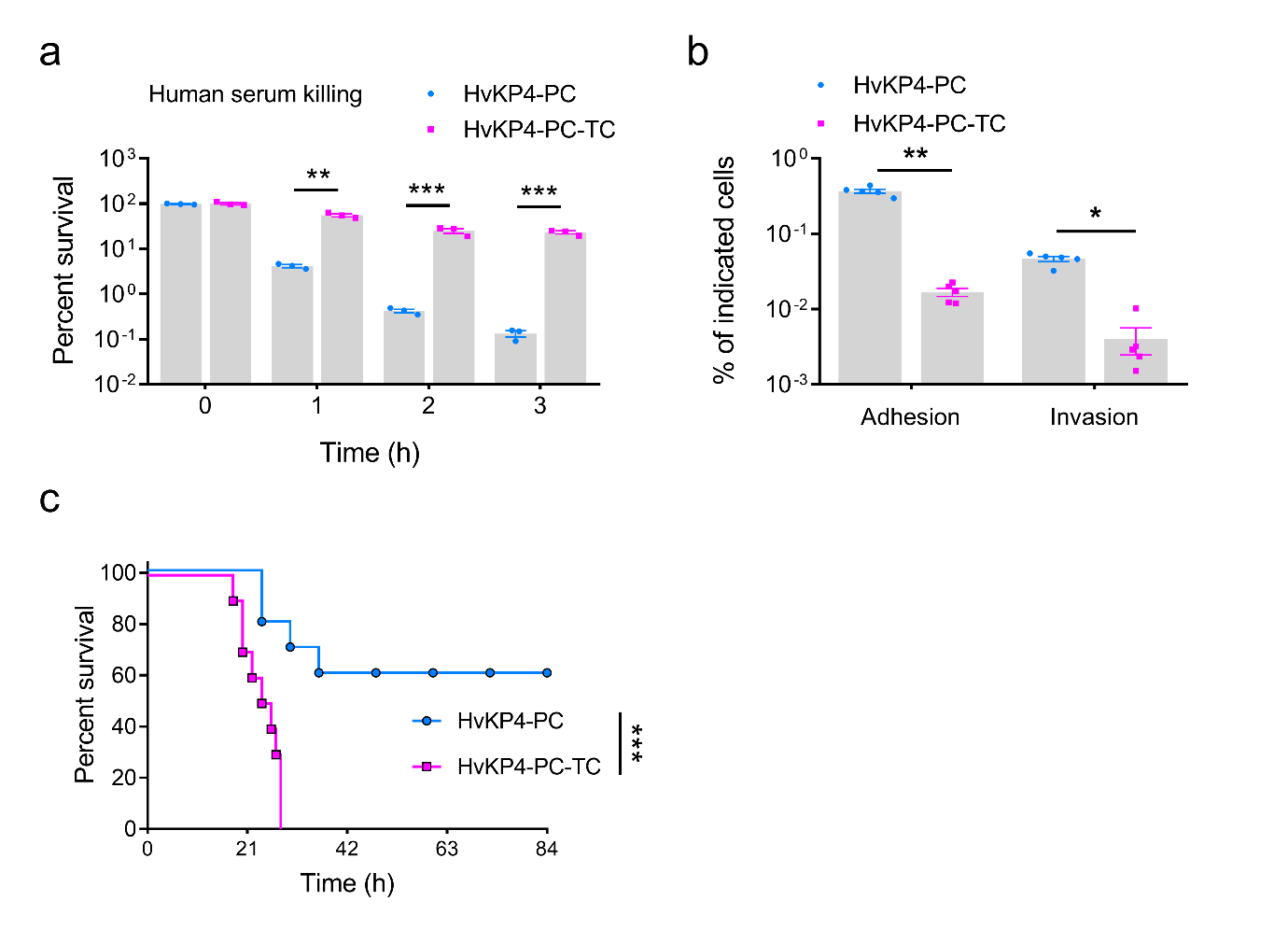
 Figure S2. The virulence potential of p16HN200-Vir in HvKP4-PC. (a)** The resistance of *Kp* strains to killing by pooled human serum. **(b)** Assay of the ability of *Kp* strains adhesion and invasion to RAW264.7 cells. Data were analyzed by one-way ANOVA test. Each data point was repeated three times. Data are presented as the mean ± S.E.M. **(c)** Virulence potential of *Kp* strains in the mouse bacteremia infection model infected with 5 × 10^7^ of the test strains. The survival of mice infected by each test *Kp* strain is depicted. The test strains included HvKP4-PC and HvKP4-PC-TC. n = 10. A log-rank (Mantel-Cox) test conducted for the survival curves revealed significant differences. * *p* < 0.05; ** *p* < 0.01; *** *p* < 0.001.

**
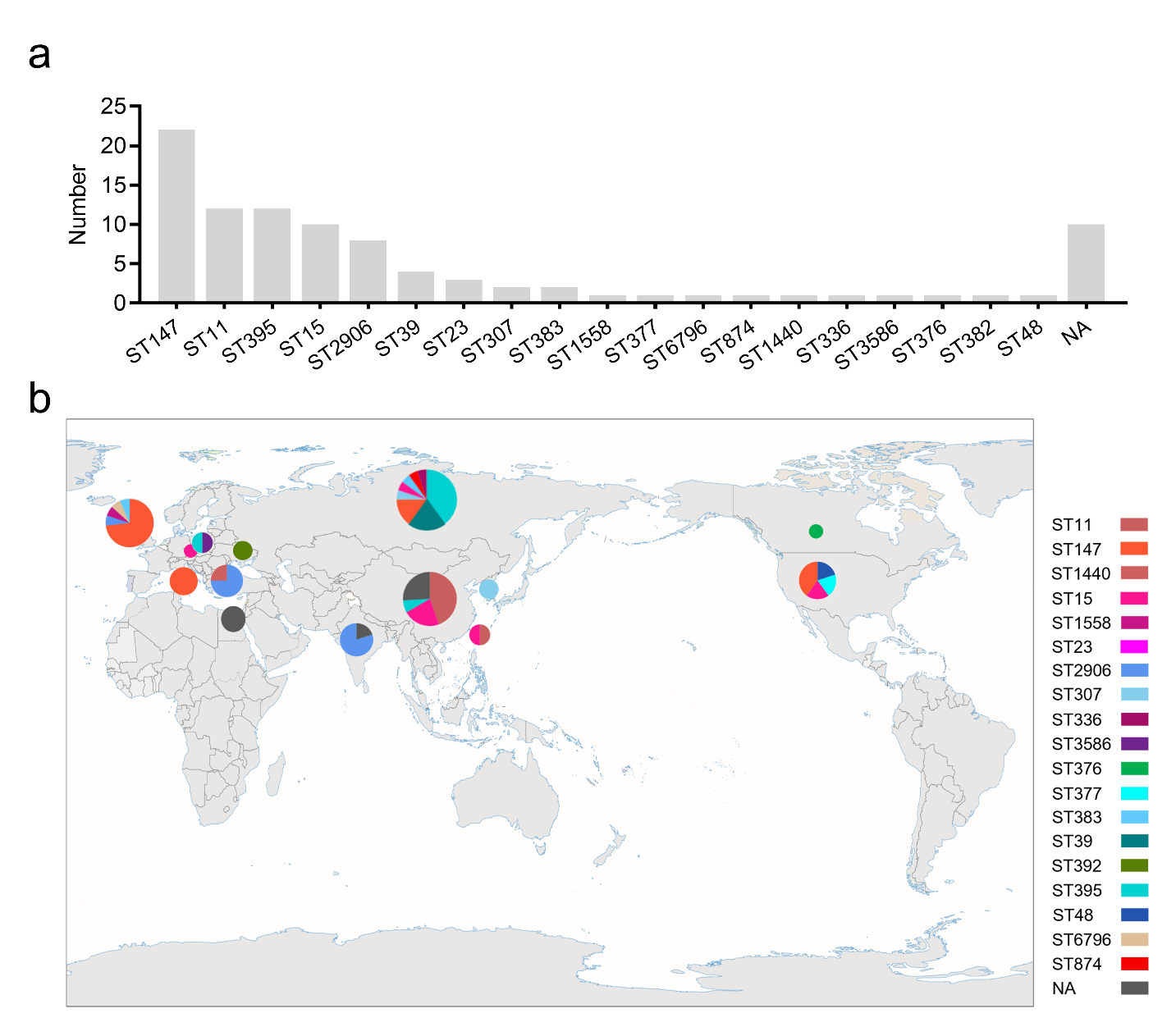
**

**Figure S3. Sequence type and geographical distribution of complete pVir-MDRs. (a)** Sequence type distribution of pVir-MDRs. **(b)** Worldwide distribution of pVir-MDRs. Different colors of sectors represent different sequence types of the host *Kp* of pVir-MDRs as shown in the right of the figure. All pVir-MDRs reported up to February 20th, 2024, were included. Pie graph areas are relatively proportional to the total number of pVir-MDR containing strains reported in each country/region.

**
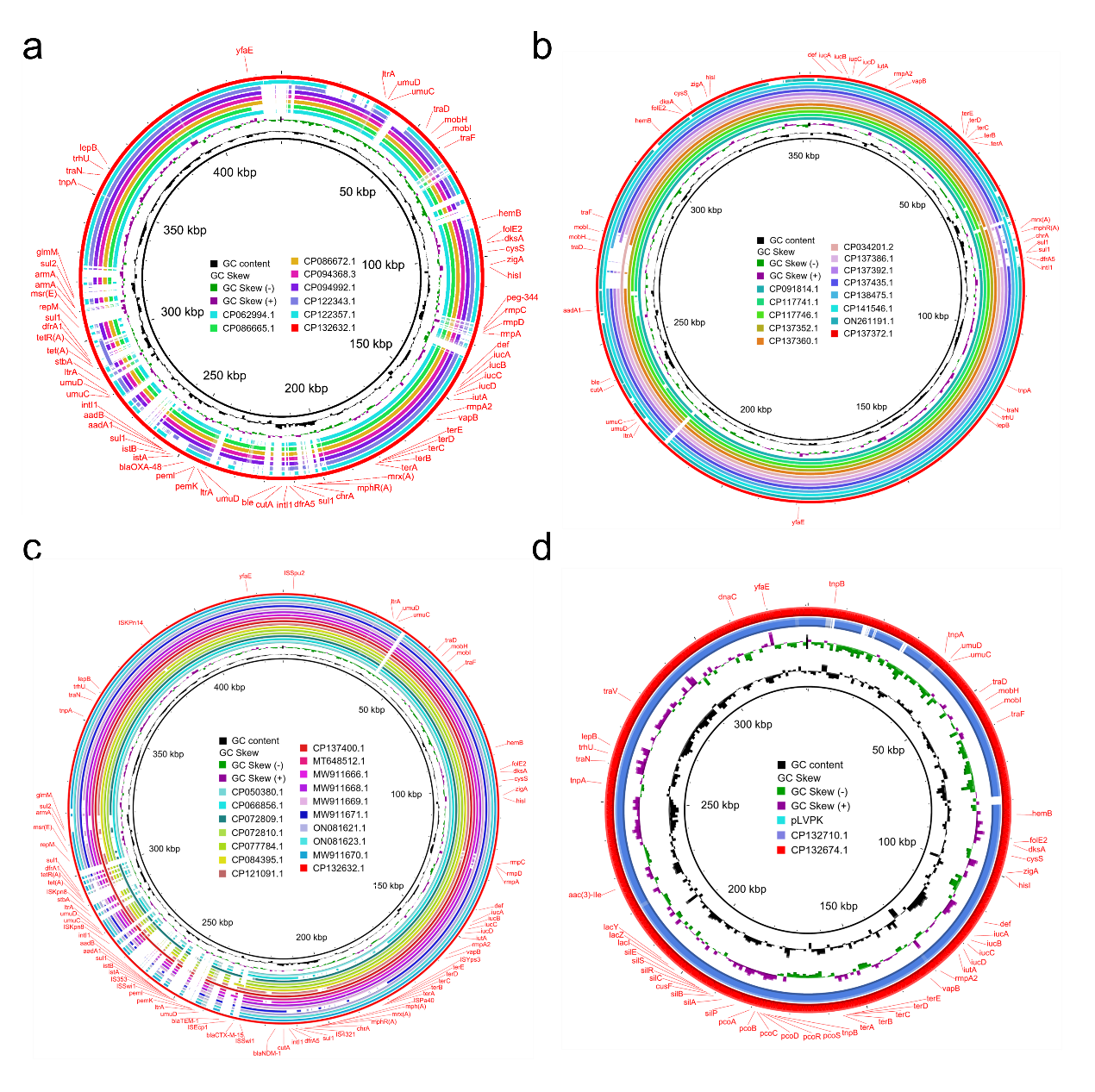
 Figure S4. Alignment of pVir-MDRs with *bla*_OXA-48_ (a), *bla*_NDM-5_ (b), *bla*_NDM-1_ (c) and the two plasmids recovered in 2005 (d, CP132710.1 and CP132674.1) by BRIG.**
